# Arthroscopic surgery or exercise therapy for degenerative meniscal lesions: a systematic review of systematic reviews

**DOI:** 10.1007/s12306-022-00760-z

**Published:** 2022-09-03

**Authors:** M. Rotini, G. Papalia, N. Setaro, P. Luciani, M. Marinelli, N. Specchia, A. Gigante

**Affiliations:** 1 Department of Clinical and Molecular Science, School of Medicine, Università Politecnica Delle Marche, Via Tronto, 10/A, 60126 Ancona, Italy; 2 Clinic of Adult and Paediatric Orthopaedic, Azienda Ospedaliero-Universitaria, Ospedali Riuniti Di Ancona, Via Tronto 10/ 60126, Ancona, Italy

**Keywords:** Arthroscopy, Degenerative, Knee, Meniscectomy, Meniscus, Placebo

## Abstract

**Background:**

Arthroscopic partial meniscectomy (APM) is widely applied for the treatment of degenerative meniscal lesions in middle-aged patients; however, such injury is often associated with mild or moderate osteoarthritis and has been reported by MRI in asymptomatic knees. Previous studies suggested, in most patients, a lack of benefit of surgical approach over conservative treatment, yet many controversies remain in clinical practice. Our aims were to assess the functional and pain scores between exercise therapy and arthroscopic surgery for degenerative meniscal lesions and to evaluate the methodological quality of the most recent systematic reviews (SRs).

**Methods:**

Two authors independently searched PubMed and Google Scholar for SRs comparing the outcome (in knee pain and functionality) of arthroscopic treatment and exercise therapy or placebo for degenerative meniscal lesions. The timeframe set was from 2009 to 2019 included.

**Results:**

A total of 13 SRs were selected. Two reviewers independently assessed the methodological quality of each paper using the AMSTAR 2 tool: seven scored as “moderate,” four obtained a “low” grade while the remaining two were evaluated as “critically low.” SRs agreed that in middle-aged patients with degenerative meniscal lesions arthroscopic surgery appears to grant no long-term improvement in pain and function over exercise therapy or placebo.

**Conclusions:**

Conservative treatment based on physical therapy should be the first-line management. However, most SRs revealed subgroups of patients that fail to improve after conservative treatment and find relief when undergoing surgery. In the future, randomized controlled trials, evidence should be looked for that APM can be successful in case of the unsatisfactory results after physical therapy.

## Introduction

The meniscus has a critical role in stress reduction and load distribution of the knee joint [1–3]. Meniscal lesions represent the most common injury of the knee and can severely affect patients’ quality of life. They can either be traumatic—associated with a knee injury, manifesting with sudden pain [4]—or result from age-related degenerative changes in the fibrocartilage tissue [5]. Such a distinction is usually made through a careful analysis of patients’ clinical history [6] and MRI patterns [7] and can also be confirmed on a histological level [8]. Degenerative meniscal lesions evolve slowly and present an age-related increase in prevalence [9]. Such lesions are often found in knees with osteoarthritis (OA) [10] and suspected to be simply one of the many manifestations of knee degeneration [11], thus identifying the real source of symptoms is sometimes very challenging. Moreover, MRI studies confirmed that this kind of injury is very common in asymptomatic middle-aged patients, consequently questioning the clinical significance of such findings [12–14]. The use of arthroscopic partial meniscectomy (APM) for the treatment of symptomatic traumatic tears is well established [15–17]; on the other hand, the best approach to degenerative meniscal lesions has been heavily discussed in the recent literature. Despite the debate and the well-known connection of meniscus removal with OA and its progression [18], arthroscopic debridement and APM have been increasingly performed in middle-aged patients [19], with no evidence of a substantially increased prevalence of meniscus tears in this population [20]. Middle-aged patients with mild or minimal signs of OA and degenerative meniscal pathology might experience symptoms relief (most notably regarding pain and function) both undergoing arthroscopic debridement and with physical therapy. The primary aim of this study was to assess the short- and long-term functional outcomes and pain scores between arthroscopic surgery and exercise therapy for the treatment of degenerative meniscal lesions in middle-aged patients. The secondary aim was to evaluate the methodological quality and summarize the results of the most recent systematic reviews on surgical or conservative treatments for degenerative meniscal lesions.

## Materials and methods

### Literature search strategy

Review methods were defined as a protocol during the study conceptualization. The literature search was performed in December 2019, based on the PubMed (including Medline) and Google Scholar electronic databases, selecting studies published from January 2009 to December 2019 and including only reviews, systematic reviews (SRs) and meta-analyses. The following search strings were used: ("middle aged"[MeSH Terms] OR ("middle"[All Fields] AND "aged"[All Fields]) OR "middle aged"[All Fields] OR ("middle"[All Fields] AND "age"[All Fields]) OR "middle age"[All Fields]) AND (("degenerative"[All Fields] OR "degeneratively"[All Fields] OR "degeneratives"[All Fields]) AND ("meniscus"[MeSH Terms] OR "meniscus"[All Fields] OR "menisci"[All Fields])). Search results were independently evaluated by two reviewers (R.M. and P.G.) and, after selecting pertinent papers, full-text articles were obtained. Reference lists from the collected articles were also examined for any eligible study absent from the initial search results.

### Study selection

To retain a high level of evidence, only SRs were included. Non-systematic reviews and clinical trials were excluded. Again, final study selection was performed independently by two reviewers (R.M. and P.G.). Evaluation of the papers was performed searching for clinical outcome assessment in the form of pain and functionality of the knee.

#### Inclusion criteria

- English-language SRs (from January 2009 to December 2019);
- Degenerative meniscal lesions treated by arthroscopic surgery (with or without partial meniscectomy);
- Comparison of the surgical treatment to conservative care (physical therapy) or placebo;
- Evaluation of the outcome must include pain and functionality with any validated score.

#### Exclusion criteria

- Studies including acute meniscal lesions from trauma or other pathologies of the knee (septic/rheumatoid arthritis, chondropathy, ligamentous lesions, etc.).

### Data collection and analysis

Two review authors independently extracted the following data for each study: authors, year of publication, study type, level of evidence, number of included studies, total number of patients, procedures performed in intervention and in control groups, outcome measures, mean follow-up, and presence of meta-analysis.

The AMSTAR 2 tool [21] was employed to assess the methodological quality of the SRs included in the study. The analysis, consisting of 16 items and defining a SR quality as either high, moderate, low, or critically low, was performed by two reviewers (R.M. and P.G.) independently using the provided checklist tool (https://amstar.ca/Amstar_Checklist.php). Any disagreement in the result was resolved by confrontation. The results of this study are reported according to the PRISMA guidelines statements [22].

## Results

**Fig. 1:**
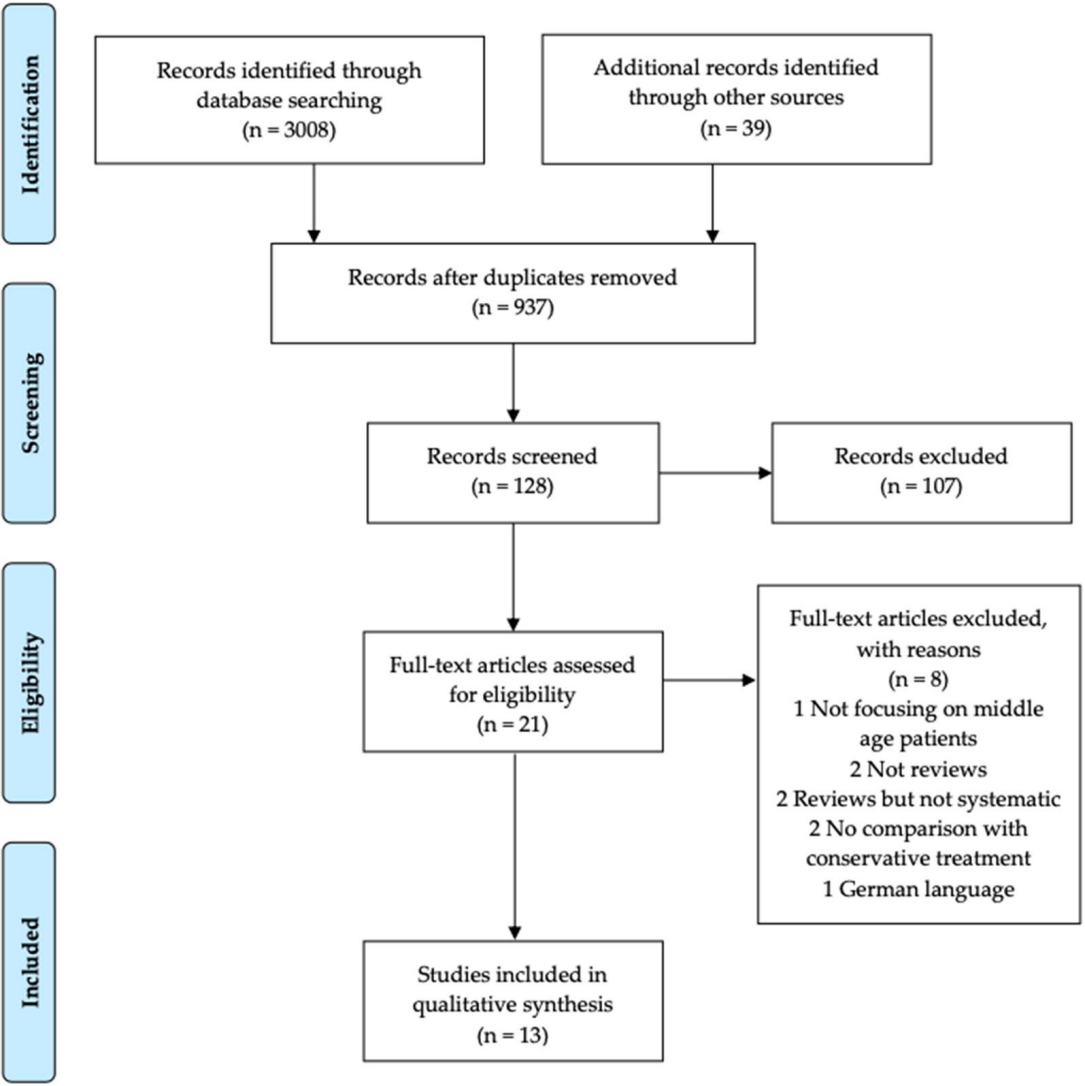
Preferred reporting items for systematic review and meta-analysis (PRISMA) flow diagram

The selection process is described in Fig. 1. PubMed searches combined provided 1728 results and Google Scholar searches combined provided 1280 results. (Duplicates between all searches were not considered at this point.) A first selection of the search results and exclusion of duplicates produced a total of 128 eligible papers. After evaluation of the abstracts and exclusion of out-of-topic studies, the full text of 21 papers was collected and examined. Once the reference list was searched for additional entries, 13 publications were selected to be included in the analysis. A complete list of the papers selected but excluded during full text examination can be found in Table 1.

**Table 1:** List of selected papers and reason for exclusion

	Author	Status	Exclusion reason
*PubMed*			
	Salata et al. [23]	Excluded	Not focusing on middle age patients, no comparison with conservative treatment
	Health quality Ontario [24]	Included	
	Katz et al. [25]	Excluded	Not a systematic review
	Khan et al. [25]	Included	
	Lamplot and Brophy [27]	Included	
	Swart et al. [28]	Included	
	Bassett et al. [29]	Included	
	Brignardello-Petersen et al. [30]	Included	
	Becker et al. [31]	Excluded	German language
	Monk et al. [32]	Included	
	Hohmann et al. [33]	Included	
	Karpinski et al. [34]	Included	
	Lee et al. [35]	Included	
*Google Scholar*			
	Petersen et al. [36]	Included	
	Ghislain et al. [37]	Excluded	Not a SR, no comparison with conservative treatment
	Thorlund et al. [38]	Included	
	van de Graaf et al. [39]	Included	
	Dias et al. [40]	Excluded	Focused on rehabilitation after meniscectomy, no conservative treatment alone involved
	Buchbinder et al. [41]	Excluded	Not a systematic review
	Stone et al. [42]	Excluded	Review but not systematic
	Azam et al. [20]	Excluded	Review but not systematic

All 13 systematic reviews included in the present study evaluated conservative therapy as control treatment, while the comparison with sham/placebo surgery was addressed in 11 systematic reviews (Table 2).

**Table 2:** Extracted data for every selected paper

Author	Year	Study type	LOE	N. of studies included	Total n. of patients	Interventions				Control				Outcome Measure	Mean Follow-up (months)	Mean Cross-over	Meta-analysis
						APM + exercise	AM	APM	AL	Exercise	Sham surgery or AL	Intraarticular Injection	Oral NSAIDs				
Health quality Ontario [24]	2014	SR	I	9 RCT	1101	4	1	4	/	6	3	/	/	KOOS, WOMAC, VAS, SF-36, Lysholm score	31.5	26.3%	No
Khan et al. [26]	2014	SR	I	7 RCT	805	/	/	7	/	5	1	1	/	KOOS, Oxford Knee, KSS, Lysholm score	/	16.2%	Yes
Petersen et al. [36]	2015	SR	I	6 RCT	940	/	/	6	/	4	1	1	/	KOOS, WOMAC, Lysholm score	22	21.4%	No
Thorlund et al. [38]	2015	SR	I	9 RCT	1270	5	/	4	/	6	3	/	/	KOOS, VAS, Lysholm score	/	19.3%	Yes
Lamplot and Brophy [27]	2016	SR	II	6 [5 RCT + 1 PS]	1037	/	1	5	/	4	2	/	/	KOOS, WOMAC, Lysholm score	15	19.3%	No
Swart et al. [28]	2016	SR	I	5 RCT	346	2	/	3	/	3	/	/	/	KOOS, VAS, Lysholm score	9	/	Yes
van de Graaf et al. [39]	2016	SR	I	6 RCT	773	3	/	3	/	5	1	/	/	KOOS, WOMAC, VAS, Lysholm score	19	22.6%	Yes
Bassett et al. [29]	2017	SR	II	9 (8 RCT + 1 PS]	1553	5	2	2	/	6	2	/	/	/	21.3	27.3%	No
Brignardello-Petersen et al. [30]	2017	SR	I	14 RCT	1665	/	/	14	/	9	3	2	/	KOOS, WOMAC, VAS, KSS, Lysholm score	/	20.1%	Yes
Monk et al. [32]	2017	SR	II	7 RCT	1201	1	/	5	1	3	3	1	/	KOOS, WOMAC, Lysholm score	/	21.8%	No
Hohmann et al. [33]	2018	SR	II	6 RCT	835	/	/	6	/	6	/	/	/	KOOS, WOMAC, Lysholm score	22.5	24.3%	No
Karpinski et al. [34]	2018	SR	I	14 RCT	1781	4	/	7	3	6	5	2	1	KOOS, WOMAC, Lysholm score	23.5	21.9%	No
Lee et al. [35]	2018	SR	I	9 RCT	1270	/	/	7	2	6	2	1	/	KOOS, WOMAC, VAS, AIMS, Lysholm score	23.2	23.7%	Yes

*APM* Arthroscopic partial meniscectomy; *AM* arthroscopic meniscectomy; *AL* arthroscopic lavage; *SR* systematic review; *RCT* randomized controlled trial; *Ps* prospective study

In total, 21 individual studies were included in the systematic reviews (Table 3).

**Table 3:** Primary studies included in systematic reviews

	Health Quality Ontario	Khan et al.	Petersen et al.	Thorlund et al.	Lamplot et al.	Swart et al.	van de Graaf et al.	Bassett et al.	Brignardello-Petersen et al.	Monk et al.	Hohmann et al.	Karpinski et al.	Lee et al.
Herrlin et al. [43]	+	+		+				+	+				
Herrlin et al. [44]	+	+	+				+	+	+	+	+	+	+
Katz et al. [45]	+	+	+	+	+		+	+	+	+	+	+	+
Kirkley et al. [46]	+			+	+			+	+			+	+
Østeras et al. [47]	+	+		+		+	+		+		+		
Østeras et al. [48]						+							
Yim et al. [49]	+	+	+	+		+	+	+	+	+	+	+	+
Hubbard et al. [50]	+											+	
Moseley et al. [51]	+			+				+	+	+		+	+
Sihvonen et al. [52]	+	+	+	+	+		+	+	+	+		+	
Vermesan et al. [53]		+	+						+	+			+
Gauffin et al. [54]			+	+	+				+		+	+	+
Chang et al. [55]				+					+			+	+
Aaron et al. [56]					+								
Merchan [57]					+							+	
Stensrud et al. [58]						+	+		+				+
Hall et al. [59]						+							
Kise et al. [19]								+	+		+	+	
Bailey et al. [60]								+					
Saeed et al. [61]									+				
Hede et al. [62]										+			

Knee arthroscopic surgery was the investigated treatment in every paper. However, techniques reported in the trials object of systematic reviews varied widely. Unspecific description such as “Arthroscopic treatment,” “Debridement,” “Meniscectomy,” and “Meniscal debridement” were commonly reported and often lacked details. Evaluation of original trials allowed to retrieve more accurate data, nevertheless unveiling different interpretation and execution of homonymous procedures:

- “Arthroscopic treatment” included lavage with either synovectomy and/or debridement and/or excision of degenerative meniscal tears and/or loose cartilage;
- “Knee arthroscopic surgery” was defined as standard procedure with meniscal resection but also any other treatment needed based on the experience of the surgeon;
- “Debridement” consisted in lavage with different amounts of saline solution and resection of loose cartilage but it might or might not include meniscus trimming/smoothing;
- “Partial meniscectomy” was most consistent with the depiction of trimming of the meniscus until solid tissue was reached, but it might or might not include the removal of loose cartilage.

The results were further confused by the association of the procedures such as in “Debridement with partial meniscectomy.”

Most of the authors reported that trials included in their reviews contained full details on rehabilitation protocols. However, similarly to the situation described for interventions, physical therapy followed very different schedules and timing between studies. Exercise, which was either supervised or unsupervised, based on home exercises or performed at the gym, showed a great variability in length and frequency such as: “supervised 1–2 times a week plus home exercises, for 6 weeks”; “twice a week for 8 weeks”; “3 supervised weeks followed by 8 weeks of home exercise program”; “twice in week 1, once in week 2 and fortnightly thereafter until week 12”; “16 supervised sessions in 8 weeks”; “for 12 weeks twice a week at home or at the gym, instructed by the therapist but without supervision”; “2–3 sessions each weeks for 12 weeks with supervision for the first week”; “3 times a week for 12 weeks monitored by a therapist.” Evaluation of the sham/placebo procedures also showed great differences in execution and naming. The reported procedures can be summarized as follows:

- “Washout” together with “Lavage” consisted of saline run through the knee in quantities ranging from 1 to 10L; however, in one study partial meniscectomy was performed if any mechanically important, unstable tear of the meniscus was encountered;
- “Simulated debridement” and “Sham surgical procedure” consisted of manipulation of the knee, request for surgical instruments by the surgeon and duration of the procedure comparable to real surgery, either with or without skin incisions;
- “Simulated partial meniscectomy” was a diagnostic knee arthroscopy without meniscectomy.

Finally, the same applies for the scores used to assess pain and functionality. The scores dedicated exclusively to pain evaluation included the visual analogue scale (VAS), Knee-Specific Pain Scale, and the pain subscale of the Knee Society Score (KSS). Specific assessment of functionality was obtained with the Tegner Activity Scale and Patient Activity Scale. The list completes with other general outcome measurement knee scores such as KOOS, Lyhsolm Knee Score, WOMET, WOMAC, EQ5D, AIMS2, SF-36, MACTAR, Oxford Knee Score, ASES, and HSS Knee Rating Score.

The mean cross-over rate refers to the percentage of patients that, at first, were enrolled in the conservative treatment group and then moved to the intervention group undergoing surgical treatment. The mean value of the trials in each SR ranges from 16.2 to 27.3%. This suggests that close to ¼ of patients undergoing physical treatment did not benefit from exercise and crossed-over to surgery during the follow-up. Khan et al. [26] reported the lowest mean cross-over rate; however, this result might be inaccurate as they assigned a value of 0% to one study [47] which did not report if any cross-over was present.

- After methodological quality assessment by AMSTAR 2, seven SRs (54%) [24, 27, 28, 33, 35, 38, 39] scored as “moderate,” four (31%) [26, 32, 34, 36] obtained a “low” grade while the remaining two (15%) [29, 30] were evaluated as “critically low.” The detailed results are reported in Table 4.

**Table 4:** AMSTAR 2 items and final results

AMSTAR 2 (16 items)																	
STUDY	PICO	A priori design	Design criteria	Literature search	Selection in duplicates	Extraction in duplicates	Excluded studies	Study detail	Risk of bias	Funding sources	Statistical comb. method	RoB in meta-analysis	Individual RoB	Heterogeneity Expl	Publications bias	Conflict of interests report	QUALITY SCORE
Health Quality Ontario [24]	YES	Partial YES	YES	Partial YES	NO	NO	NO	YES	YES	YES	NO META ANALYSIS	NO META ANALYSIS	YES	YES	NO META ANALYSIS	YES	MODERATE
Khan et al. [26]	YES	YES	NO	YES	YES	YES	NO	Partial YES	YES	NO	YES	NO	NO	YES	YES	NO	LOW
Petersen et al. [30]	YES	Partial YES	NO	Partial YES	YES	YES	NO	Partial YES	NO	NO	NO META ANALYSIS	NO META ANALYSIS	YES	YES	NO META ANALYSIS	NO	LOW
Thorlund et al. [38]	YES	YES	NO	Partial YES	YES	NO	YES	Partial YES	YES	NO	YES	YES	YES	YES	NO	YES	MODERATE
Lamplot and Brophy [27]	YES	Partial YES	NO	Partial YES	YES	YES	Partial YES	Partial YES	YES	NO	NO META ANALYSIS	NO META ANALYSIS	YES	NO	NO META ANALYSIS	YES	MODERATE
Swart et al. [28]	YES	YES	NO	Partial YES	YES	YES	NO	NO	YES	NO	YES	NO	YES	YES	NO	YES	MODERATE
van de Graaf et al. [39]	YES	YES	NO	YES	YES	YES	NO	Partial YES	YES	NO	YES	YES	YES	YES	NO	YES	MODERATE
Bassett et al. [29]	NO	NO	NO	NO	NO	NO	NO	NO	NO	NO	NO META ANALYSIS	NO META ANALYSIS	NO	NO	NO META ANALYSIS	YES	CRIT. LOW
Brignardello-Petersen et al. [30]	YES	YES	NO	YES	YES	YES	NO	Partial YES	YES/NO	NO	NO	YES	YES	NO	YES	YES	CRIT.LOW
Monk et al. [32]	YES	Partial YES	NO	Partial YES	YES	YES	YES	Partial YES	Partial YES	YES	NO META ANALYSIS	NO META ANALYSIS	NO	YES	NO META ANALYSIS	NO	LOW
Hohmann et al. [33]	YES	NO	NO	Partial YES	YES	YES	NO	Partial YES	YES	NO	NO META ANALYSIS	NO META ANALYSIS	YES	YES	YES	NO	MODERATE
Karpinski et al. [34]	YES	Partial YES	NO	Partial YES	YES	YES	Partial YES	Partial YES	NO	NO	NO META ANALYSIS	NO META ANALYSIS	YES	YES	NO META ANALYSIS	YES	LOW
Lee et al. [35]	YES	YES	NO	Partial YES	YES	YES	NO	Partial YES	Partial YES	NO	YES	YES	YES	YES	NO	YES	MODERATE

## Discussion

The aims of the study were to assess the short- and long-term functional outcomes and pain scores between arthroscopic surgery and exercise therapy for the treatment of degenerative meniscal lesions in middle-aged patients and to evaluate the methodological quality of the SRs on this topic. All the SRs we selected had to deal with the complexity of comparing results of slightly different procedures and outcome measurements, both among the operative and control groups. Such complexity was passed on when trying to summarize the details of each SR.

A meta-analysis was performed by 6 of the SRs included in the present study. The results of the meta-analysis regarding each outcome are shown in Table 5. It has been shown better improvement in the short-term functional outcomes and pain scores in favor of arthroscopic surgery. However, at long-term follow-up there were no differences in functional outcomes and pain scores between arthroscopic surgery and exercise therapy.

**Table 5:**
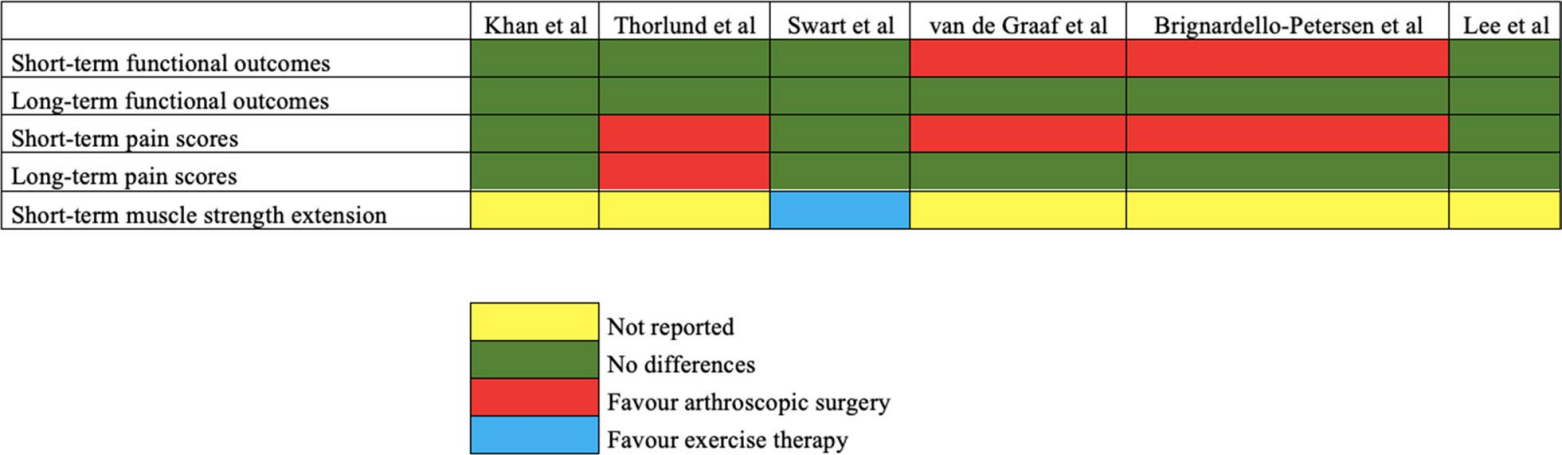
Assessment of the outcomes in the meta-analysis

Khan et al. [26] reported the results of 7 (randomized controlled trials) RCTs (1 of which included steroids injections as control group and therefore goes beyond the scope of this analysis). Based on five trials, they reported a significant improvement in short-term (< 6 months) functionality after arthroscopic meniscal debridement when compared with exercise treatment. However, there was no long-term (between 6 months and 2 years) difference in functional improvement. Pain after surgery showed no improvement over physical therapy, either in the short-term and long-term. The last trial evaluated by the authors considered sham surgery as control treatment and reported no significant difference in terms of pain and functionality. A tendency to cross-over was reported in 5 out of 7 of the RCTs, with values ranging from 0 to 30%.

Thorlund and colleagues [38] conducted an extensive systematic review based on 9 trials evaluating the results of surgical treatment. APM was the main procedure in every study, combined with debridement in 2 trials. Physical therapy was the control choice in 6 of the trials while the remaining 3 compared surgical treatment with sham surgery/lavage. The results showed a small but statistically significant advantage in terms of pain relief with interventions in comparison with control treatments, however, this difference was only present when considering 3 and 6 months of follow-up. Moreover, such difference in visual analogue scale (VAS) was considered to be below the clinically relevant improvement. Regarding physical function, the authors did not find any difference among the analyses.

In the systematic review from Swart et al. [28], among the 12 included papers, 3 trials investigated exercise therapy alone compared to meniscectomy alone while two evaluated exercise therapy after meniscectomy compared to meniscectomy alone. The results were reported as pain and function in the short (< 3 months) and long (> 3 months) term. When directly comparing exercise therapy and meniscectomy, both treatments obtained similar results in terms of pain and function, with an advantage in terms of muscle strength in the short term for the patients undergoing exercise therapy. The results of exercise therapy compared to no exercise therapy after meniscectomy were very conflicting: in terms of pain, one study reported an advantage of exercise therapy in the short term but not in the long term while the other showed no difference at any follow-up; the examined studies did not agree in terms of function either, one showing a significant advantage for the exercise group at short and long term, the other showing no difference.

Another study from van de Graaf et al. [39] analyzed 5 RCTs to evaluate the effectiveness of APM against exercise therapy (4 trials) or sham surgery (1 trial): Both groups in all included studies obtained a significant improvement compared to baseline. In conclusion, they found a statistically significant better outcome in the APM group up to 6 months of follow-up in terms of function as well as in terms of pain; however, the benefit was gone at 1 year of follow-up.

Brignardello-Petersen et al. [30] performed an update on the topic with an extensive and detailed SR and meta-analysis. After evaluation of 14 RTCs (9 compared APM with exercise therapy and 2 with sham surgery; 1 paper focusing on arthroscopic lavage and 2 on intraarticular injection will not be discussed) the authors concluded that while APM provides a benefit in pain and function over physical therapy in the short term, this difference is small and non-persistent, narrowing up on the long term.

Finally, Lee et al. [35] conducted a systematic review of the literature and meta-analysis in 2018. Of the 9 RCTs having arthroscopic surgery as treatment, 6 compared it with exercise therapy and two with placebo/close needle lavage. The last trial evaluated steroid injections as control therapy and does not apply to the topic of this study. The authors concluded that both in terms of pain and functionality, arthroscopic surgery offered no significant advantage over conservative exercise treatment.

The remaining seven SRs did not perform a meta-analysis, reporting that it was not possible due to the high heterogeneity across study populations, interventions, and outcomes.

The analysis from the Ontario Health Technology Assessment Series [24] evaluated a total of nine RCTs in which operative treatment was compared to exercise alone (6 trials) and to sham surgery (3 trials). In their conclusions, they reported no significant difference in pain and functionality between patients who received arthroscopic debridement (with or without meniscectomy) and patients treated with physical therapy or sham surgery. Among the trials, the authors reported a percentage of patients that crossed over from the control to the operative group due to unsatisfactory results, ranging from 0 to 30%.

The study from Petersen et al. [36] analyzed 5 RCTs on APM, 4 of which showed no difference between the results of arthroscopic partial removal of the meniscus and control treatment (3 trials on physical therapy and 1 on sham surgery). However, the last trial reported that patients treated with APM had significantly less pain at mid- and final follow-up (3 and 12 months) than those following exercise therapy. The authors highlighted a rate of switch from the physiotherapy group to the intervention group ranging between 6.6% and 34.9% among 5 RCTs.

In 2016 Lamplot and Brophy [27] reviewed 5 RCTs and 1 prospective cohort study relevant for this topic. While 2 of the papers reported there was no benefit in arthroscopic surgery over exercise therapy, 2 others supported the operative treatment and 1 detailed that it varied based on OA severity. The last RCT found no advantage in surgical treatment compared to placebo. In spite of some limitations, the authors concluded that patients with degenerative knee changes and degenerative meniscal tear can experience symptoms improvements with arthroscopic meniscectomy, especially in cases with mild OA or not responsive to conservative treatment. However, they also added that since most patients included in the trials showed clinically significant improvements after conservative treatment, physical therapy should be carried forward prior to operative intervention, especially in cases already displaying moderate OA. Again, the authors reported an amount of crossover between groups (6–30%).

In 2017 Bassett et al. [29] conducted a review on this topic including nine papers. APM was confronted with exercise therapy in seven studies, while the comparison was made with sham surgery in two studies. Every paper examined by the authors returned no significant difference in the outcome between APM and control groups; however, one study reported greater benefit from APM in selected populations such as those with BMI > 30 kg/m^2^ or no/mild signs of OA. According to the authors, approximately 1/3 of the patients in the control group were not satisfied and crossed over to the operative group reaching results equivalent to the rest of the patients.

In their research, Monk et al. [32] collected both RCTs and SRs looking for evidence, in a study design similar to the present one (although SRs were mostly focused on meniscal repair/transplant). Related to meniscal resection, they identified 8 papers (7 RCTs and 1 SR). Of the 7 RCTs, 3 compared APM with physical therapy while 2 compared it with sham surgery. (The last 2 trials evaluated total meniscectomy and steroids injections which go beyond the scope of this paper.) The one SR comparing APM with physical therapy was already included in the present study and discussed above [26]. When compared with physical therapy there was no difference in the outcome of APM. In a similar way, the improvement in the group of sham surgery was not different from the group treated with APM. The reported crossing over was as high as 35% in one study [36], with outcomes comparable to the patients initially randomized to the exercise and operative group, respectively.

Hohmann et al. [33] included only level I and II studies in their SR and quantitative synthesis, with a special attention for evidence quality. After researching the literature, they selected 6 RCTs that compared APM with physical exercise. The conclusion of the authors was that, due to the high risk of bias affecting all studies, giving a conclusive answer was impossible and both approaches were to be considered viable for treating the condition. This paper also reported a tendency to cross-over in four studies (between 10 and 30%), considered among the causes of bias.

Karpinski et al. [34] conducted a systematic review to compare the effects of arthroscopic surgery and exercise therapy in patients with knee OA, including those with degenerative meniscal lesions. The selection resulted in 14 RCTs, five of which contained either intra-articular injections, joint lavage or NSAIDs administration alone and are therefore excluded from the discussion. Of the remaining nine articles, six included various combinations of exercise therapy while the other three placebo.

After highlighting various limitations of recent trials, the authors concluded the evidence supports that patients with knee OA have no benefit after arthroscopic surgery. However, they identified subgroups which might benefit from it, one being people with non-traumatic flap tears of the medial meniscus. Out of 14, five papers reported a cross-over component, with the highest represented by Katz et al. [45] and Herrlin et al. [44], respectively, at 34.9% and 27.7%.

Most of the aforementioned SRs agree that arthroscopic surgical approach brings no benefit over physical therapy (and placebo) when treating degenerative meniscal lesions in middle-aged patients. The exceptions are represented by Lamplot and Brophy which reported conflicting results and by Hohmann et al. which invite to caution when interpreting the results of the trials due to their quality and bias. However, this analysis presents some intrinsic limitations that requires critical evaluation of the results. First, some RCTs were included in more than one systematic review object of this paper. This situation is expected and common among SRs given their nature but can lead to bias due to repetition of results. Another source of error comes from the great heterogeneity in treatment definition, as highlighted in the results section. Therefore, the results drawn by the literature are based on a very variable and generic definition of knee arthroscopic surgery. Comparably, control treatment suffers from the same lack of standardization. Most notably, surgical treatment does not seem to provide long-lasting improvements despite the inconsistency among exercise programs. Future evidence could improve this area by adopting standardized and dedicated protocols [63]. Finally, placebo procedures ranged as well, exhibiting the same problem despite being much less represented than physical therapy. Medical therapy did not seem to be regulated either among the intervention nor the control groups of the various studies. Another important source of bias originated from the tendency to cross-over from the control to the intervention group, exceeding 30% of patients in some trials. In such subgroups, physical treatment failed and patients obtained satisfactory results only after surgery. Despite the confounding factor, this situation is close to a real-world scenario and might represent a subgroup of patients that, after unsuccessful conservative treatment, benefit from arthroscopic surgery. However, such finding can also be interpreted as resulting from a perceived “missed opportunity”: patients informed about the possibility of surgical treatment but allocated to physical therapy group might result less satisfied with residual symptoms and therefore seek surgery. A distinction between these opposite hypotheses is relevant since knee arthroscopy has been linked to the onset or worsening of knee OA [18] and the risk of undergoing total knee arthroplasty following arthroscopic surgery significantly increases with age [64], and therefore, it should be limited to those patients with appropriate surgical indications.

All these limitations display in the fact that only seven of them were able to obtain a “moderate” score from a dedicated and validated tool for evidence quality assessment such as AMSTAR 2. Such a result, when considering the unambiguous verdict of most of SRs, outlines an evidence that can be considered reliable but should seek improvements in the future.

## Conclusions

The present review of recent systematic reviews suggests that in middle-aged patients with degenerative meniscal lesions, arthroscopic surgery by partial meniscectomy and debridement grants no long-term improvement in pain and function over exercise therapy or placebo. However, in the short term it has been proved a small significant difference in pain relief and functionality in favor of the surgical approach. Nevertheless, such improvements applied to the short-term and did not last in the long term. Based on these results, a conservative treatment consisting of physical therapy should be the first-line management for degenerative meniscal tears in middle-aged patients. However, the majority of reviews described a subgroup of patients that failed to improve with conservative treatment and found relief when undergoing surgery. Therefore, evidence suggests that APM might be justified and decisive in case of unsatisfactory results after physical therapy, and future RCTs should focus on further evaluating this hypothesis. These conclusions rely on moderate/low methodological quality evidence, which also suffers from confounding factors such as a lack of standardized treatments and controls across trials. Future evidence could benefit from standardization of treatments such as a surgical technique focused on APM without additional surgical gestures and control groups adhering to registered rehabilitation protocols.

## Data Availability

Data is available in the paper.
